# Changes in arterial flow velocity and pulsatility following endarterectomy for symptomatic high degree carotid artery stenosis: insights from the Carotis7T Study

**DOI:** 10.1016/j.cccb.2025.100517

**Published:** 2025-11-05

**Authors:** Carolijn J.M. de Bresser, Ellen van Hulst, Elisabeth C. van der Voort, Simone J.A. Donners, Marjolijn L. Rots, Raechel J. Toorop, Gert J. de Borst, Jaco J.M. Zwanenburg

**Affiliations:** a Department of Vascular Surgery, University Medical Center Utrecht, Heidelberglaan 100 3584 CX, Utrecht, the Netherlands; b Translational Neuroimaging Group, Center for Image Sciences, University Medical Center Utrecht, Utrecht, the Netherlands; c Board of Directors, Reinier de Graaf Gasthuis, Reinier de Graafweg 5 2625 CE, Delft, the Netherlands

**Keywords:** Carotid artery stenosis, Carotid endarterectomy, Cerebral small vessels, Cerebral perforating artery flow, Pulsatility, 7 Tesla MRI

## Abstract

**Objective:**

Patients with symptomatic high degree carotid artery stenosis often exhibit a reduced perfusion pressure in the cerebral vasculature in absence of sufficient collaterals. The aim of this research was to evaluate changes in mean blood flow velocity and pulsatility within the cerebral perforating arteries following carotid endarterectomy (CEA).

**Methods:**

Fifteen patients with symptomatic high degree (>50 %) carotid artery stenosis were included in the monocentre prospective observational Carotis7T study. All patients underwent 7T magnetic resonance imaging (MRI) one day prior to CEA and three months postoperative. Mean blood flow velocity (Vmean) and pulsatility index (PI) were measured in the first segment of the middle cerebral artery (M1 of MCA), and in the perforating arteries of the basal ganglia (BG) and semi-oval centre (CSO), using a 2D phase-contrast 7T MRI sequence. Outcomes on the ipsilateral side were compared between the preoperative and postoperative situation.

**Results:**

A postoperative increase (+14.2 %) in Vmean and (+10.7 %) in PI was seen at the level of the MCA. A slight decrease in Vmean and a slight increase in PI were observed at both the level of the BG (respectively -5.6 % and +12.8 %) and CSO (respectively -13.2 % and +11.0 %) between the preoperative and postoperative situation following CEA. However, these changes were not statistically significant.

**Conclusion:**

In this small single centre patient sample, our findings suggest that revascularization by removal of the stenosis and atherosclerotic plaque does not result in a measurable impact on the brain’s perfusion at BG and CSO level at three months post intervention.

## Highlights

• Improved optimal medical therapy (OMT) and surgical experience in carotid interventions have reduced perioperative stroke after carotid endarterectomy (CEA). The 2017 and 2023 European Society of Vascular Surgery guidelines propose imaging criteria to identify patients on OMT who may benefit from CEA. Although symptomatic status affects perioperative diffusion-weighted imaging lesions and cerebral perfusion, specific predictive imaging markers for late stroke remain unclear. Advances in ultra-high field 7T magnetic resonance imaging enable *in vivo* assessment of blood flow velocity and pulsatility in small perforating brain arteries, offering potential for novel risk markers for stroke.

## Introduction

Cerebral blood flow in small arteries is kept stable by the vasomotor response in healthy individuals, however, this protective mechanism may fail in elderly people or people suffering from cerebrovascular disease [1,2]. Emerging evidence suggests that blood flow dynamics diverge from average patterns in patients with vascular pathologies [3,4]. Patients with unilateral or bilateral extracranial carotid artery stenosis often exhibit hemodynamic abnormalities in the cerebral vasculature, including a reduced perfusion pressure when intracerebral collateral circulation is insufficient [5,6]. Previous studies have shown that patients with asymptomatic occlusion of the internal carotid artery (ICA) have a better preserved hemodynamic status of the brain compared with symptomatic patients with a stenosed carotid artery [[7], [8], [9], [10]]. Carotid endarterectomy (CEA) is the recommended treatment for patients with symptomatic significant (> 50 %) carotid artery stenosis to restore cerebral blood flow and prevent future stroke [11]. Carotid artery stenting (CAS) is an alternative for patients unsuitable for CEA and in younger patients [12]. Hemodynamic disturbances contribute to approximately 50 % of perioperative strokes following CEA, playing a pivotal role in the diverse pathogenesis of postoperative stroke [13,14]. Cerebral hyper perfusion syndrome (CHS) is a relatively rare (0 – 3 %), potentially devastating complication leading to stroke [14,15]. Transcranial doppler (TCD) is the golden standard for monitoring intra-operative changes in the blood flow of the middle cerebral artery (MCA), thereby helping to reduce cerebral hyper perfusion related morbidity and mortality following CEA [15,16].

Advances in ultra-high field magnetic resonance imaging (MRI) comprising the 7 Tesla (7T) scanner, have enabled, precise, *in vivo* measurement of blood flow velocity (Vmean) and pulsatility index (PI) within the brain’s small perforating arteries, providing a valuable tool for assessing the cerebral microcirculation [17,18]. Prior research has shown that carotid occlusive disease may be associated with abnormal blood flow in cerebral perforating arteries across a broad patient cohort, including those with significant carotid artery stenosis not eligible for CEA, as well as those with an occlusion of the internal carotid artery (ICA) [4]. While recent studies have compared flow abnormalities between these affected patients and the healthy population, no research to date has examined the hemodynamic impact of CEA on the relationship between pre- and postoperative cerebral blood flow dynamics and pulsatility in the small brain vessels [4,19]. This study aims to address the existing gap by utilizing 7T MRI 2D phase-contrast imaging to evaluate changes in blood flow velocity and pulsatility within the cerebral microcirculation in symptomatic patients with ipsilateral significant carotid artery stenosis undergoing CEA.

## Materials and methods

### Carotis7T study and study population

The data used in this study originates from the Carotis7T Study, a monocentre prospective observational study at the University Medical Center Utrecht. Approval of the medical ethical committee (NedMec) has been obtained (NL 63,925.041.18) and the study has been conducted in accordance with the declaration of Helsinki [20]. Patient recruitment took place between December 2021 and September 2024. All patients were discussed in a multidisciplinary team and CEA procedures were performed according to national and international guidelines [11]. Included patients were compatible for MRI and had a symptomatic carotid artery stenosis of at least 50 % according to the North America Symptomatic Carotid Endarterectomy Trial (NASCET) criteria [21]. All included participants provided written informed consent, after which 3T and 7T brain MRI were performed one day prior to surgery and three months after surgery. Preoperative baseline measurements were used as control condition, allowing each patient to act as their own control. Symptomatic disease was defined as a transient ischemic attack (TIA), stroke or amaurosis fugax within 6 months prior to surgical intervention. Patients with contraindications for MRI, previous CEA or carotid artery stenting (CAS) in the artery to be treated, recent revascularization of the contralateral carotid artery, vertebral artery or intracranial artery carried out within 6 weeks prior to CEA, recent coronary artery bypass grafting within 3 months prior to inclusion or other major surgery within 6 weeks of inclusion were excluded.

### Outcomes

Our primary outcome is the change in Vmean (cm/s) and PI (defined as [Vmax – Vmin]/Vmean) on the stenotic side before and after CEA within the cerebral microcirculation, measured in the first segment of the MCA (M1), and in the perforating arteries in the basal ganglia (BG) near the periventricular white matter and in semi-oval centre (CSO) near the deep white matter.

### MRI data acquisition and processing

**Fig. 1:**
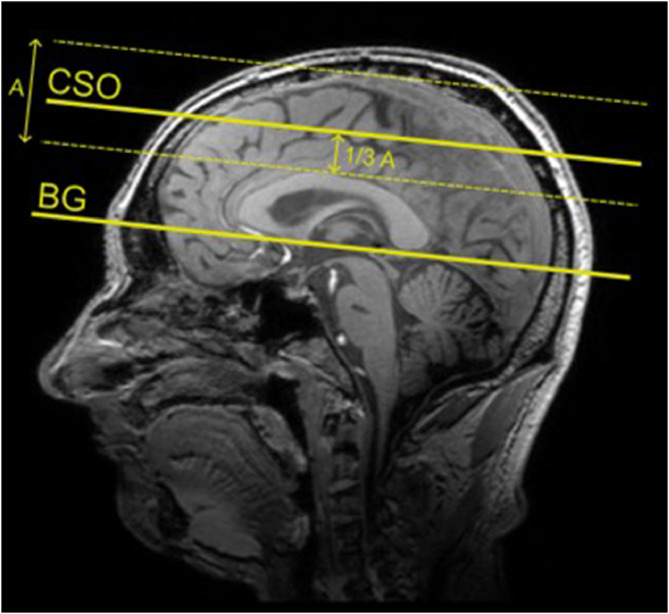
Planning of the 2D PC scans for the BG and CSO was performed using a T1-weigthed image as reference. For the BG 2D PC scan, the acquisition plane was aligned parallel to the inferior margin of the corpus callosum, with the imaging slice positioned through the anterior commissure. For the WM 2D PC scan, the acquisition plane was oriented similar to that of the BG scan. The imaging slice was positioned approximately one-third of the distance from the superior margin of the corpus callosum to just below the skull.

Participants underwent 7T brain MRI (Philips Healthcare, Best, The Netherlands) using a 32-channel receive head coil with a quadrature transmit coil (Nova Medical, MA). 2D phase-contrast (PC) acquisitions were performed to measure Vmean and PI in the M1 of both the left and right MCAs, as well as in the small perforating arteries in the BG and CSO (Table 1) [17,18]. The planning of the M1 was performed by positioning the imaging plane perpendicular to the M1 based on a T1-weighted image. To ensure consistent positioning between the pre- and postoperative scans, the pre-operative planning was used as reference for the post-operative planning. Slice planning and orientation of the 2D PC scans for the BG and CSO scans are shown in Fig. 1. The acquisitions were time-resolved over the cardiac cycle, with 0.3 × 0.3 mm^2^ resolution and a velocity encoding (Venc) of 20 cm/s for the BG and 4 cm/s for the CSO scan. Slice planning of the 2D PC acquisition was based on a T1-weighted image. A peripheral pulse oximeter was used for retrospective gating. Additionally, a 3D fluid-attenuated inversion recovery (FLAIR), a pseudo-continuous arterial spin labelling (PCASL) and a T1-weighted scan were acquired at 3T

**Table 1:** Imaging parameters of the 2D phase-contrast acquisitions in the first segment of the middle cerebral artery, basal ganglia and semioval center.

	M1	BG	CSO
FOV (mm) (RLxAP)	250 × 250	250 × 180	250 × 250
Slices	1	1	1
Acquired voxel size (mm) (RLxAPxFH)	0.5 × 0.5 × 3	0.3 × 0.3 × 2	0.3 × 0.3 × 2
Reconstructed voxel size (mm) (RLxAPxFH)	02 × 0.2 × 3	0.2 × 0.2 × 2	0.2 × 0.2 × 2
Flip angle (°)	50	50	65
Venc (cm/s)	120	20	4
TR (ms)	17	28	29
TE (ms)	4	15	17
TFE factor	2	2	2
SENSE factor (AP direction)	2	1	1.5
Scan time (min:s)*	01:56	04:37	04:17

⁎ For a cardiac frequency of 65 bpm AP: anterior-posterior; BG: basal ganglia; bpm: beats per minute; CSO: semioval centre; FH: feet-head; FOV: field of view; M1: first segment of the middle cerebral artery; min: minutes; mm: millimetres; ms: milliseconds; s: seconds; RL: right-left; TE: echo time; TFE: turbo field echo; TR: repetition time; Venc: encoded velocity.

The 2D PC images were processed using the Small vessEL MArker (SELMA) open-source analysis software to obtain velocity and pulsatility measures [22]. First, 2D PC images were scored on motion artifacts and excluded if motion artifacts were extensive, spreading over the region of interest (ROI), or if the artifacts caused visible instability of the background phase over the cardiac cycle. Artifact scoring was performed by EH and borderline cases were scored in consensus with JZ. M1 flow velocity was defined as the average over the contoured lumen, as identified by the SELMA algorithm, using Vmean and magnitude values across the cardiac cycle that were significantly above the noise level. Subsequently Vmean and PI were derived. Perforating arteries were assessed in two regions of interest, using a manual delineation in the BG and a 2D white matter (WM) mask at the CSO. Detection and analysis of the perforating arteries in the ROIs was performed as previously published [17,23,24]. In the BG, the algorithm identified perforating arteries based on pixels exhibiting a Vmean and magnitude over the cardiac cycle that were significantly above the noise level. A manual selection of the identified vessels was performed by CB and borderline cases were scored in consensus with JZ. Selection was based on two criteria: 1) assessment of the circularity of the detected vessel to remove non-perpendicular vessels to the imaging plane, and 2) by removing detections in a close distance from each other, where the artery with the highest velocity was kept. Segmentation of the WM mask was performed using the Computational Anatomy Toolbox (CAT12, version 2550) an extension of Statistical Parametric Mapping (SPM12, version 7771, Welcome Trust Centre for Neuroimaging, London, UK) using a probability threshold of >0.95 and registration was performed using the FMRIB Software Library (FSL, version 6.0, The University of Oxford, UK) [25]. Subsequently, only the central WM was selected using a whole brain mask eroded with 80 voxels as template. Lastly, clear ghosting artefact were manually removed from the mask. In the CSO, only a significant Vmean over the cardiac cycle was used for identification of the vessels. The algorithm automatically excluded ghosting zones, non-perpendicular vessels and vessels closers than 6 pixels apart from each other, as these were predominantly false detections corresponding to larger vessels or those with non-perpendicular orientations. Masks for the BG and CSO were split into a stenotic/revascularized and contralateral mask [17,23,24]. For each detected vessel, the velocity trace was extracted over the entire cardiac cycle. Vmean and PI, based on the normalized velocity curves, were assessed by averaging over all detected cerebral perforating arteries within the ROI [17,24]. Probability maps of white matter hyperintensities (WMH) were generated using the lesion growth algorithm [26] as implemented in the Lesion Segmentation Tool toolbox (version 3.0.0 - www.statistical-modelling.de/lst.html) for SPM12, with the pre-operative FLAIR and T1-weighted images as input. WMH volumes were subsequently calculated using a 50 % probability threshold [26]. Cerebral blood flow (CBF) values from pre- and postoperative ASL scans were estimated using the BASIL toolbox within FSL (oxford_asl), applying default quantification settings and an M0 image was used for calibration [27]. Structural images were preprocessed using FLS (v6.0.7.17) [28]. 3T T1-weighted images were segmented and registered to ASL space using FSL software to obtain gray matter CBF values.

### Statistical analysis

Baseline characteristics were summarized as mean (standard deviation [SD]) for continuous, normally distributed variables and median (interquartile range [IQR]) for skewed continuous variables, and absolute numbers with proportions for categorical variables. Normality of data was tested using the Shapiro-Wilk test. Next, we determined whether statistically significant differences existed in Vmean and PI between pre- and postoperative measurements, with paired samples *t*-tests. Logarithmic and square root transformation applied to pairs of variables that were not normally distributed were not used, as the clinical interpretation of the results would be hampered following transformation. Statistical significance was considered with a two-sided p-values of <0.05. All statistical analyses were performed using R Statistical Software version 4.2.1 (R foundation, Vienna, Austria).

## Results

**Fig. 2:**
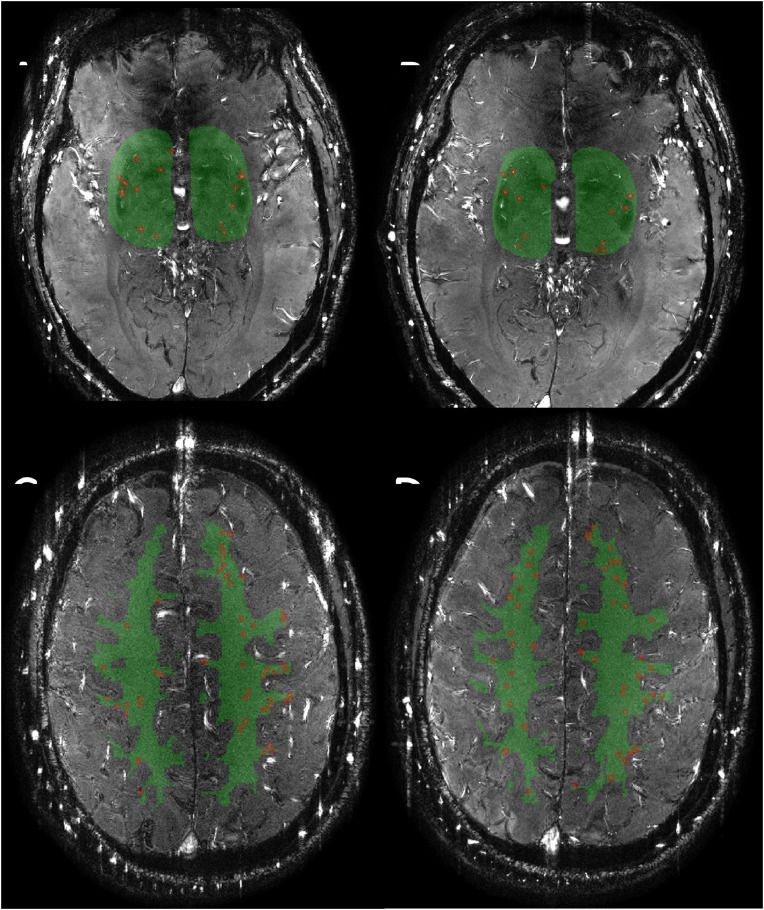
Two-dimensional (2D) PC images in the basal ganglia (BG, A and B) and semioval center (CSO, C and D) for the pre-operative (A, C) and post-operative (B, D) situation for one subject. The ROIs are highlighted in green. The included vessels in the analysis, as detected by the algorithm and after manual selection for the BG are highlighted in red. For visualization purposes, the detected vessels are dilated with a disk-shaped structuring element with a radius of 6 pixels.

Baseline characteristics of the Carotis7T cohort are presented in Table 2. Fifteen of 36 (42 %) patients successfully underwent 7T MRI scans preoperatively and postoperatively. Fig. 2 shows a representative example of the detected vessels pre- and post-operatively. The cohort was a typical atherosclerotic cohort, consisting of a male majority, with a mean age of 70 years, predominantly comprising patients with hypertension, using anticoagulants and/or statins and former or current smokers. The average preoperative modified ranking scale (mRS) was 1. All patients, except one who had a narrow or non-existent temporal bone window, underwent CEA with TCD measurement. No cerebrovascular events, cardiovascular events or deaths occurred within the 30-day postoperative period. Due to subject movement during scanning, a few scans were excluded because of poor image quality, with exclusion rates of 6.7 % preoperatively and 13.3 % postoperatively for the level of the BG and 13.3 % preoperatively and 26.7 % postoperatively for the level of the CSO.

**Table 2:** Baseline characteristics of the Carotis7T cohort.

Variable	N = 15^1^
Age, years	69.3 ± 8.3
Male sex	12/15 (80 %)
Vascular risk factors	
Hypertension	8/15 (53 %)
Diabetes	2/15 (13 %)
Smoking status	
Current smoker	1/15 (7 %)
Former smoker	9/15 (60 %)
Hypercholesterolemia	7/15 (47 %)
Statin use	13/15 (87 %)
History of cardiovascular event	5/15 (33 %)
History of peripheral arterial disease	2/15 (13 %)
Use of anticoagulants	14/15 (93 %)
Type of symptoms	
Amaurosis fugax	3/15 (20 %)
Single cerebral TIA	4/15 (27 %)
Multiple cerebral TIAs	3/15 (20 %)
Minor stroke	2/15 (13 %)
Major non-disabling stroke	3/15 (20 %)
Degree of ipsilateral stenosis	
50–69 %	1/15 (7 %)
70–99 %	14/15 (93 %)
WMH volume (ml)	3.15 [2.31 – 6.81]^2^

1 Summary statistics: Mean (SD) for normally distributed variables, and n/N ( %).

2 Median (IQR) for skewed variable TIA: Transient Ischemic Attack.

### First segment of the MCA

**Fig. 3:**
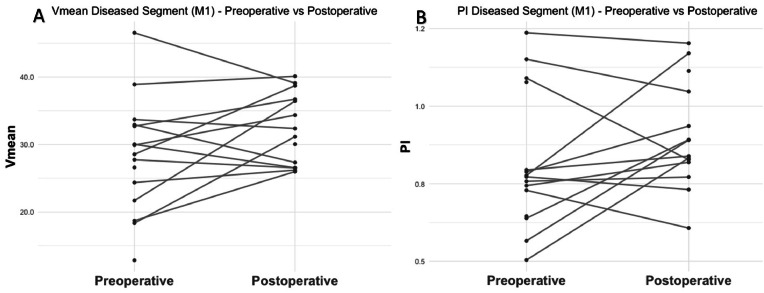
Overview of the preoperative and postoperative relative increase at the level of the M1 for the mean flow velocity (A) and pulsatility index (B).

M1: first segment of the middle cerebral artery; PI: pulsatility index; Vmean: mean blood flow velocity.

The mean Vmean at the stenotic side at the level of the M1 preoperatively and postoperatively were 28.2 ± 8.5 and 32.3 ± 5.3, respectively (Fig. 3A). This 14 % increase in mean velocity was not statistically significant (*p* = 0.16, 95 % CI: −1.30 – 7.05). The mean PI at the stenotic side increased by 11 %, from 0.82 ± 0.22 preoperatively to 0.91 ± 0.17 postoperatively. This change was statistically not significant (*p* = 0.20, 95 % CI: −0.04 – 0.18) (Fig. 3B).

### Basal ganglia

**Fig. 4:**
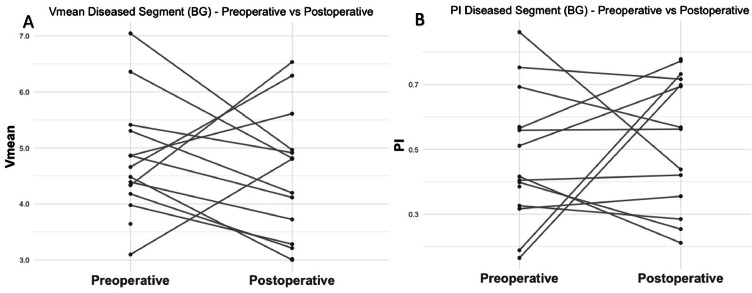
Overview of the preoperative and postoperative relative change in mean blood flow velocity (A) and pulsatility index (B), at the level of the basal ganglia.

BG: Basal ganglia; PI: pulsatility index; Vmean: mean blood flow velocity.

The mean Vmean at the stenotic side at the level of the basal ganglia preoperatively and postoperatively were respectively 4.7 ± 1.0 and 4.5 ± 1.2 (Fig. 4A). However, this 5.6 % decrease was not statistically significant (*p* = 0.50, 95 % CI: −1.10 – 0.56). The mean PI at the stenotic side were 0.47 ± 0.20 and 0.53 ± 0.20 for the preoperative and postoperative situation, and this 13 % increase was also not statistically significant (*p* = 0.59, 95 % CI: −0.12 – 0.21, Fig. 4B). A non-significant reduction in the number of detected vessels was observed on the stenotic side between preoperative and postoperative assessment, decreasing from 6.7 ± 3.1 to 5.4 ± 1.9 (*p* = 0.21, 95 % CI: −4.24 – 1.01).

### Semi-oval centre

**Fig. 5:**
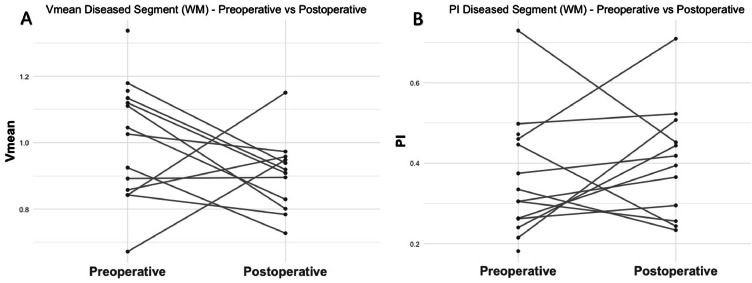
Overview of the preoperative and postoperative relative change in mean blood flow velocity (A) and pulsatility index (B) at the level of the semi-oval centre.

PI: pulsatility index; Vmean: mean blood flow velocity; WM: white matter.

The mean Vmean on the stenotic side at the semi-oval centre preoperatively and postoperatively were 1.0 ± 0.2 and 0.9 ± 0.1, respectively (Fig. 5). However, this 13 % decrease in Vmean was not statistically significant (*p* = 0.28, 95 % CI: −0.25 – 0.08). The mean PI at the stenotic side were 0.36 ± 0.15 and 0.40 ± 0.14 for the preoperative and postoperative situation, respectively, and this 11 % increase was also not statistically significant (*p* = 0.51, 95 % CI: −0.08 – 0.14, Fig. 3B). A non-significant reduction in the number of detected vessels on the stenotic side was observed, decreasing from 23 ± 12 to 16.8 ± 6.4 (*p* = 0.35, 95 % CI: −8.46 – 3.29).

An overview of the mean outcomes for the Vmean and PI at the M1, BG and CSO in both the preoperative and postoperative situation is provided for the revascularized side and contralateral side in Table 3. The change between preoperative (35.6 ± 11.7 ml/100 *g*/min) and postoperative (39.2 ± 10.5 ml/100 *g*/min) gray matter CBF was not significant (*p* = 0.23).

**Table 3:** Mean velocity and pulsatility outcomes in the first segment of the middle cerebral artery, basal ganglia and the white matter.

Variable	Middle cerebral artery		Basal Ganglia		Semi-oval centre	
	Mean	SD	Mean	SD	Mean	SD
Mean blood flow velocity (Vmean)						
Preoperative on the revascularized side	28.2	8.5	4.7	1.0	1.0	0.2
Postoperative on the revascularized side	32.3	5.3	4.5	1.2	0.9	0.1
Preoperative on the contralateral side	30.1	8.7	4.5	1.1	1.1	0.2
Postoperative on the contralateral side	30.9	5.4	5.0	2.3	0.9	0.2
Pulsatility Index (PI)						
Preoperative on the revascularized side	0.82	0.22	0.47	0.20	0.36	0.15
Postoperative on the revascularized side	0.91	0.17	0.53	0.20	0.40	0.14
Preoperative on the contralateral side	0.88	0.18	0.48	0.25	0.46	0.14
Postoperative on the contralateral side	0.88	0.18	0.49	0.21	0.50	0.19

PI: pulsatility index; SD: standard deviation; Vmean: mean blood flow velocity.

## Discussion

In this exploratory study, we measured cerebral blood flow velocity and pulsatility, measured at the levels of the MCA, BG and CSO, before and after CEA in patients with symptomatic carotid artery stenosis. The measured Vmean increased at the level of the M1, decreased at both the BG and CSO and the measured PI was higher at all levels. However, none of these changes were statistically significant.

The observed values for BG and CSO perforating artery Vmean and PI in our patient population is of a similar magnitude compared to previous findings in elderly and patients with carotid artery stenosis. Across all participants, a gradual decline in both Vmean and PI was found from the MCA toward the CSO, indicating a reduction in blood flow parameters toward deeper brain structures [4,17]

The observed nonsignificant increase in Vmean at the level of the MCA postoperatively, is in line with previous literature [29]. Hemodynamic alterations at the MCA level lead to cerebral hyper perfusion in 13.2 % and cerebral hyper perfusion syndrome in 3.5 % of the patients undergoing CEA [30]. The accompanying nonsignificant increase in PI is likely related to the decrease in inflow resistance, which reduces the amplitude of the reflection wave, after removal of the carotid stenosis [29,31]. This slight increase in PI was also observed at the levels of the perforating arteries, indicating that the hemodynamic effects of CEA may extend beyond large arteries and influence the cerebral microcirculation. In contrast, a decrease in Vmean was noted in these vessels, although this finding requires confirmation in a larger cohort. This opposite effect on the Vmean in the perforating arteries compared to the MCA may suggest that different autoregulatory mechanisms are at play in the microvasculature compared to larger cerebral arteries, possibly reflecting the presence of smooth muscle-mediated vasomotor regulation in larger arteries. If confirmed, the underlying mechanism remains uncertain and may be related to regional differences in vascular reactivity, autoregulatory adjustments, or metabolic demand [32].

Interpretation of these hemodynamic parameters is challenging, as multiple vascular changes occur simultaneously following revascularization. Postoperatively, a decline in the number of perforating arteries is observed at both the BG and CSO levels [4,19]. Similar trends have been reported in other vascular systems, such as cardiac collaterals, where regression occurs during long-term follow-up [33,34]. Additionally, studies in Moyamoya patients have demonstrated a reduction in leptomeningeal collaterals following cerebral revascularization, accompanied by an increase in total hemispheric blood flow [33]. Collateral development capacity is influenced by age, with older individuals exhibiting reduced collateral formation in large vessel occlusion. This age-related collateral rarefaction is characterized by a decline in collateral number and diameter, as well as increased vessel tortuosity [35,36]. The observation may also reflect generally larger diameters of perforating arteries before CEA, as autoregulatory vasodilation in response to carotid stenosis reduces vascular resistance and increases vessel caliber, thereby enhancing their detectability on MRI [37]. Most included vessels are similar to or smaller than the voxel size, particularly in the BG and CSO regions where mean velocity is used as a threshold, making smaller diameters more prone to partial volume effects that reduce velocity readouts and lower detectability [17,18]. Although different vessels may be included pre- and postoperatively, consistent thresholding based on physiological properties across time points ensures that averaged values still reflect the overall hemodynamic state of this category of vessels. Future studies could optimize acquisition and co-registration methods to enable repeated detection of the same vessels, allowing more detailed assessment of microvascular adaptation after CEA.

Gray matter CBF was comparable to those of age-matched populations and showed no significant postoperative change [38]. This suggests that, despite regression of collaterals and alterations in perforating artery morphology, cortical perfusion is preserved, likely reflecting compensatory autoregulatory mechanisms at the tissue level. The median WMH volume in our cohort was 3.1 mL, which is consistent with normative median values for adults aged 60–80, indicating a modest burden of small vessel disease [39]. While we did not analyse WMH in relation to cerebral hemodynamics, such lesions may influence flow velocity and pulsatility in perforating arteries supplying deep brain structures[4,40]

When comparing our findings to a cohort of patients with unilateral carotid artery stenosis exceeding 80 % up to complete carotid occlusion, we observed generally higher Vmean and PI [4]. A possible explanation for this discrepancy may be attributed to the inclusion of patients with carotid occlusion within the latter cohort, whereas carotid occlusion served as an exclusion criterium and is therefore absent in our study population. In individuals with complete carotid occlusion and an anatomically intact circle of Willis or sufficient collaterals, the contralateral ICA can compensate by supplying blood to the affected hemisphere, possibly explaining the lower Vmean and PI [41].

Given that hemodynamic disturbances, characterized as intra- or postprocedural bradycardia, asystole or any hypotension requiring treatment, account for nearly 50 % of ischemic strokes in a large Dutch cohort with recently symptomatic carotid artery stenosis, early detection and prevention is of critical importance [14]. In the context of our small cohort, our findings did not reach statistical significance, but they may reflect early adaptive responses in cerebral hemodynamics.

Our study has certain limitations. First, the small penetrating arteries analysed in this study are close to the detection limits of 7T MRI. Most of the included vessels have diameters comparable to or smaller than the voxel size, making them particularly susceptible to partial volume effects. These effects typically result in an overestimation of the PI and an underestimation of Vmean, possibly contributing partly to the observed (non-significant) decrease post-operatively [17]. However, these effects are present in both the pre-and postoperative images, which allows at least to observe relative changes. Second, few scans were excluded due to poor image quality resulting from subject movement during scanning (BG: 6.7 % preoperatively, 13.3 % postoperatively and CSO: 13.3 % preoperatively and 26.7 % postoperatively). Additionally, motion may have affected the included scans, potentially reducing their quality. This limitation was also reported in other studies and can be explained by our relatively older population with a higher tendency to move during MRI scanning than younger people [17,42]. Various strategies can be employed to reduce motion effects, including acquisitions that track and correct motion or techniques that monitor motion during scanning, which can then be statistically controlled during analysis [43]. Third, methodological variations may have influenced the results. Although the planning followed a predefined protocol, some degrees of freedom remained unavoidable. This could affect the pre- and postoperative planning and associated variability in the measured values, since planning was performed manually by multiple experience operators (JZ, EV, EH). Lastly, our study was limited by a relatively small sample size of fifteen patients. The imaging protocol, which included a 30-minute 3T MRI scan followed by a 60 – 75-minute 7T MRI scan, along with the fact that patients recently experienced a stroke within 14 days of undergoing CEA, likely contributed to a high rate of participant opt-out (58 %). Future studies could focus on selecting only the most essential 7T sequences, improving patient preparation and comfort, with features such as better table padding or audiovisual distraction to make participation more tolerable, especially in patients with recent brain infarction. The aforementioned limitations, combined with the inevitable limited signal-to-noise ratio in these small vessels, may have prevented us from observing any differences in Vmean or the PI at the level of the MCA, BG and CSO in our study.

## Conclusion

In our cohort of patients with symptomatic carotid artery stenosis undergoing CEA, we observed no significant changes in Vmean and PI at the level of the M1, BG and CSO. These findings suggest that, in this small sample, surgical removal of carotid stenosis did not result in a measurable impact on brain perforating artery flow at all levels. However, subtle trends were noted, and in a larger cohort, these hemodynamic parameters may provide more definitive insights into the cerebrovascular effects of carotid revascularization and the underlying pathophysiology of carotid artery disease.

## Funding

This publication was partly financed by the Dutch Research Council (NWO, project number 18,674).

## What this paper adds

Improved optimal medical therapy (OMT) and surgical experience in carotid interventions have reduced perioperative stroke after carotid endarterectomy (CEA). The 2017 and 2023 European Society of Vascular Surgery guidelines propose imaging criteria to identify patients on OMT who may benefit from CEA. Although symptomatic status affects perioperative diffusion-weighted imaging lesions and cerebral perfusion, specific predictive imaging markers for late stroke remain unclear. Advances in ultra-high field 7T magnetic resonance imaging enable *in vivo* assessment of blood flow velocity and pulsatility in small perforating brain arteries, offering potential for novel risk markers for stroke.

## CRediT authorship contribution statement

**Carolijn J.M. de Bresser:** Writing – review & editing, Writing – original draft, Visualization, Software, Project administration, Investigation, Formal analysis, Data curation, Conceptualization. **Ellen van Hulst:** Writing – review & editing, Writing – original draft, Validation, Software, Project administration, Investigation, Formal analysis, Data curation. **Elisabeth C. van der Voort:** Writing – review & editing, Investigation, Data curation. **Simone J.A. Donners:** Writing – review & editing, Investigation, Data curation. **Marjolijn L. Rots:** Writing – review & editing, Methodology. **Raechel J. Toorop:** Writing – review & editing. **Gert J. de Borst:** Writing – review & editing, Supervision, Resources, Methodology, Funding acquisition, Conceptualization. **Jaco J.M. Zwanenburg:** Writing – review & editing, Validation, Supervision, Resources, Methodology, Funding acquisition, Formal analysis, Conceptualization.

## Declaration of competing interest

The authors declare the following financial interests/personal relationships which may be considered as potential competing interests: Jaco J.M. Zwanenburg reports financial support was provided by University Medical Centre Utrecht. If there are other authors, they declare that they have no known competing financial interests or personal relationships that could have appeared to influence the work reported in this paper.
